# Human Microbiota in Esophageal Adenocarcinoma: Pathogenesis, Diagnosis, Prognosis and Therapeutic Implications

**DOI:** 10.3389/fmicb.2021.791274

**Published:** 2022-01-14

**Authors:** Wanyue Dan, Lihua Peng, Bin Yan, Zhengpeng Li, Fei Pan

**Affiliations:** ^1^Department of Gastroenterology and Hepatology, The First Medical Center, Chinese PLA General Hospital, Beijing, China; ^2^Medical School of Nankai University, Tianjin, China

**Keywords:** microbiota, esophageal adenocarcinoma, Barrett’s esophagus, gastroesophageal reflux disease, microbial therapy

## Abstract

Esophageal adenocarcinoma (EAC) is one of the main subtypes of esophageal cancer. The incidence rate of EAC increased progressively while the 5-year relative survival rates were poor in the past two decades. The mechanism of EAC has been studied extensively in relation to genetic factors, but less so with respect to human microbiota. Currently, researches about the relationship between EAC and the human microbiota is a newly emerging field of study. Herein, we present the current state of knowledge linking human microbiota to esophageal adenocarcinoma and its precursor lesion—gastroesophageal reflux disease and Barrett’s esophagus. There are specific human bacterial alternations in the process of esophageal carcinogenesis. And bacterial dysbiosis plays an important role in the process of esophageal carcinogenesis *via* inflammation, microbial metabolism and genotoxicity. Based on the human microbiota alternation in the EAC cascade, it provides potential microbiome-based clinical application. This review is focused on novel targets in prevention, diagnosis, prognosis, and therapy for esophageal adenocarcinoma.

## Introduction

Esophageal cancer (EC) is the seventh most common cancer with an estimated 604, 000 new cases worldwide in 2020. It is also the sixth leading cause of cancer death with an estimated 544,000 deaths in 2020 (Sung et al., 2021). The age-standardized 5-year net survival of the EC patients was in the range of 10–30% between 2010 and 2014, except in Japan and Korea. In many countries, the age-standardized 5-year net survival trends increased by 6–10% from 2000 to 2014 (Allemani et al., 2018). Tackling the global burden of the EC is one of the major challenges in this century.

There are two main distinctive histological subtypes that account for more than 95% of EC, esophageal adenocarcinoma (EAC) and esophageal squamous cell carcinoma (ESCC). Generally, ESCC occurs in the upper two-thirds of the esophagus, whereas EAC typically occurs in the lower third of the esophagus (Arnold et al., 2020). In recent decades, the incidence rate of EAC in the United States has increased to 7.2 per 100,000 populations, while the incidence rate of ESCC has been sharply decreasing (Simard et al., 2012). From 1999 to 2008, the incidence rate of EAC showed an increasing trend in all races, except for American Indian or Alaska native, whose average annual percent change was −0.1. Although increased progressively during 1992 through 2007, 5-year relative survival rates for EAC were poor (Simard et al., 2012). Gastroesophageal reflux disease (GERD) and obesity have been identified as strong risk factors for EAC. Tobacco smoking and alcohol consumption might facilitate EAC development. In contrast, weight loss, estrogens, dietary fiber, and vegetable intake might protect against its development (Coleman et al., 2018). These risk factors provided clues for the primary prevention of EAC, thus public health interventions to modify them are advisable (Lagergren and Lagergren, 2013; Thrumurthy et al., 2019). Our knowledge of the human microbiota has expanded exponentially with the development of novel molecular methods, especially metagenome sequencing. Much of the current literature on cancer pays particular attention to the human microbiota (Plottel and Blaser, 2011). Accumulating evidence suggests that human microbiota contributes to colorectal cancer, gastric cancer, liver cancer, lung cancer and breast cancer (Schwabe and Jobin, 2013). Besides, the human microbiota is widely regarded as a potential co-factor for the development of EAC and its precursor Barrett’s esophagus (BE) (Peters et al., 2017; Quante et al., 2018).

Herein, we present the current state of knowledge linking human microbiota to esophageal adenocarcinoma, with a primary focus on its potential clinical applications.

## Human Microbiota

The human genetic makeup is virtually identical. Different from the human genome, the metagenome of the human microbiome shows greater variability (Lloyd-Price et al., 2016). The human microbiota is a highly individual, complex, and dynamic community in each healthy individual (Consortium, 2012; Gilbert et al., 2018). There are 10–100 trillion symbiotic microorganisms and 500–1000 species of bacteria in the human body, whereas the number of sub-species could be far more (Turnbaugh et al., 2007). Even in the same person, it will be extraordinarily different from before. Besides, there are diverse archaea, fungi, and viruses colonizing in the human body, although the current understanding of them remains limited. The digestive tract is the largest microbial habitat in the human body, which has the largest number of microbes and the most kind of species (Gupta et al., 2017). The gastrointestinal microbiota has three main ways of colonization: in the epithelial mucosa, in digest particles, and suspension solution (Dominguez-Bello et al., 2019). Investigators have been devoted to identifying the core microbiota, which is characterized by a group genera of being found in all populations regardless of their geographical location, ethnic background or residence. A population-level analysis reported a 14-genera core microbiota (*Lachnospiraceae*, *Ruminococcaceae*, *Bacteroides*, *Faecalibacterium*, *Blautia*, *Roseburia*, *Erysipelotrichaceae*, *Coprococcus*, *Dorea*, *Clostridiaceae*, *Hyphomicrobiaceae*, *Clostridiales*, *Veillonellaceae*, *Clostridium* XIVa) by assessing human fecal samples (Falony et al., 2016).

Given the well-established carcinogenesis that *Helicobacter pylori* had in gastric cancer and human papillomavirus had in cervical cancer, human microbiota was starting to be considered as a key factor that influences both human health and disease in the past decade (Bashan et al., 2016). Along with the deep-going of the research, in addition to special pathogens, the imbalance of normal microbiota can also cause diseases, such as allergy and psoriasis. Studies in colon cancer animal models have revealed evidence for tumor-promoting effects of the microbiota dysbiosis. There is a significant decrease in the number of tumors with the treatment of wide-spectrum antibiotics (Schwabe and Jobin, 2013). In addition, microbial diversity is associated with disease status. It is well established that type 2 diabetes and inflammatory bowel disease have low intestinal microbial diversity, as well as cervical intraepithelial neoplasia and bacterial vaginosis have high vaginal microbial diversity (Fredricks et al., 2005; Consortium, 2012; Qin et al., 2012; Mitra et al., 2015; Proctor, 2019). The mechanisms by which the human microbiota is involved in carcinogenesis primarily includes inflammation, immunity, metabolism, genomic integration, and genotoxicity (Scott et al., 2019). As an example, Gram-negative bacteria could acquire carcinogenic ability by producing genotoxin (He et al., 2019). Consequently, Microbiome Wide Association Studies, including DNA sequencing, metabolomics, proteomics, and computation, are providing potential microbiome-based screening tools, diagnostic markers, and adjuvant therapies (Kåhrström et al., 2016). It links microbial community structure and metabolites with disease status, which will lead clinical researches to a new field in the future.

## Human Microbiota Alternation in the Esophageal Adenocarcinoma Cascade

### Esophageal Dysbiosis in the Esophageal Adenocarcinoma Cascade

Unlike the oral cavity, stomach, or intestine, the esophagus has its unique microbiota. A total of 41 genera belonging to six phyla of bacteria colonizing in the normal distal esophageal were identified (Pei et al., 2004). Six phyla consisted of *Firmicutes*, *Bacteroides*, *Actinobacteria*, *Proteobacteria*, *Fusobacteria*, and TM7. And top five genera were *Streptococcus*, *Prevotella*, *Veillonella*, *Rothia*, and *Megasphaera*. Furthermore, shotgun sequencing identified that there were not only abundant bacteria but also a relatively low abundance of viruses and eukaryotes in the esophagus, such as betaherpesvirus 7 and *Candida glabrata* (Deshpande et al., 2018). The esophageal microbiota is classified mainly into three main community types, and it has been proved significant differences across the three types. Among them, the predominant genus is *Streptococcus* in type 2 and it is *Prevotella* in type 3. Type 1 is an intermediate type between type 1 and type 2, which is composed of not only *Streptococcus* and *Prevotella*, but also increased abundances of *Haemophilus* and *Rothia* (Deshpande et al., 2018). Although there is no statistical difference in the total amount of microbial DNA among normal esophagus, reflux esophagitis (RE), and BE, the microbial communities are different among them. By detecting bacterial populations of the distal esophagus, the percentage of Bacteroidetes in the normal esophagus, RE, and BE increased successively, but the percentage of Proteobacteria was detected successively (Liu et al., 2013). The normal esophageal mucosa had higher levels of Gram-positive *Firmicutes* and *Actinobacteria* compared to RE, BE, and EAC (Zhou et al., 2020). The microbe composition of esophagus samples including low-grade dysplasia (LGD), high-grade dysplasia (HGD), EAC, and healthy controls, were analyzed by 16S DNA sequencing. The top five different microbial taxa in abundance at the phylum level were *Firmicutes*, *Proteobacteria*, *Bacteroidetes*, *Actinobacteria*, and *Fusobacteria*. Compared with controls, phylum *Planctomycetes* and genus *Balneola* were decreased across disease groups, especially in HGD and EAC. And phylum *Crenarchaeota* was similarly decreased (Peter et al., 2020). The influence of age, host genetics and disease status on the esophageal microbiome has been identified. In support of these findings, a prospective study showed age was positively associated with the relative abundance of *Streptococcus* and negatively associated with relative abundance of *Prevotella melaninogenica* by using amplicon sequencing from 106 subjects. Deshpande et al. (2018) (Elliott et al., 2017) demonstrated a connection between host genetics and the composition of the esophageal microbiome with the help of MicrobiomeGWAS. Although the disease did not affect the global taxonomic composition of the esophageal microbiome, increasing Gram-negative bacteria taxa were found in esophageal carcinogenesis, which was only appearing in the disease states (Deshpande et al., 2018).

In order to avoid the interference of microorganisms in other parts of the digestive tract, investigators put forward various methods. At earlier stages of research on esophageal microbial colonization, esophageal biopsy and aspiration specimen measurement were applied in analyzing esophageal microbial composition. By Yang’s preliminary statistics of previous cultivation-independent studies on esophageal microbiota, the number of bacterial species detected by biopsy samples ranged from 7 to 166 (Yang et al., 2014). And they found enrichment of *Streptococcus* on esophageal microbiota. In another research, a total of 18 species were isolated from normal esophageal mucosa, while only three genera were detected in esophageal aspirate specimens, including *Lactobacilli*, *Streptococci*, and yeasts (Macfarlane et al., 2007). For patients with Barrett’s esophagus, the highest relative proportions were *Anaerococcus*, *Streptococcus*, and *Alloicoccus* in the esophagus, while the highest relative proportions were *Fusobacterium*, *Prevotella*, and *Dialister* in the uvula (Okereke et al., 2019). Recently, the microbial communities of EAC samples were examined by means of Cytosponge. EAC tissues had decreased microbial diversity, including a reduction of Gram-positive taxa (*Granulicatella*, *Atopobium*, *Actinomyces*, and *Solobacterium*) as well as Gram-negative taxa (*Veillonella*, *Megasphaera*, and *Campylobacter*) compared with healthy controls (Elliott et al., 2017). These studies confirmed that decreased microbial diversity and altered microbial composition may play a significant role in the EAC cascade.

### Oral Dysbiosis in the Esophageal Adenocarcinoma Cascade

**FIGURE 1:**
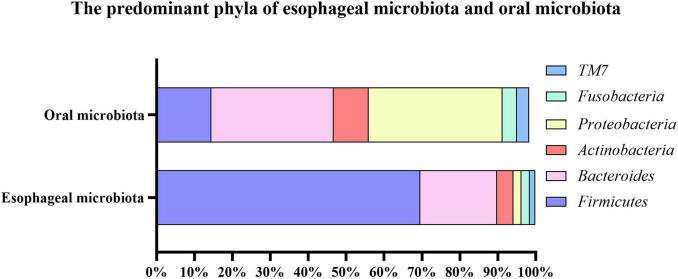
The predominant phyla of esophageal microbiota and oral microbiota. The top six most abundant phyla of esophageal microbiota consisted of *Firmicutes* (69.60%), *Bacteroides* (20.20%), *Actinobacteria* (4.30%), *Proteobacteria* (2.20%), *Fusobacteria* (2.20%), *TM7* (1.40%); And the top six most abundant phyla of oral microbiota consisted of *Proteobacteria* (35.34%), *Bacteroides* (32.20%), *Firmicutes* (14.48%), *Actinobacteria* (9.26%), *Fusobacteria* (3.76%), *TM7* (3.25%).

The oral cavity is the initial part of the digestive tract. It consists of oral lips, cheek, palate, teeth, tongue, and salivary gland. Microorganisms inhabit the available surface of oral cavity, such as the surfaces of teeth, tongue and mucosal membranes (Lamont et al., 2018). Thus, polymicrobial communities which inhabit the oral cavity have unique biogeography. The Human Microbiome Project (HMP) collected the specimens of 15 to 18 body sites from over 200 individuals. Seven of body sites were taken from the mouth including buccal mucosa, keratinized attached gingiva, hard palate, saliva, tongue and two surfaces along with the tooth. Segata et al. (2012) analyzed sub-gingival plaques, supra-gingival plaques, stool and oral specimens from the HMP. They demonstrated that the microbial communities of the tongue are similar to saliva and the microbial communities of buccal mucosa are similar to keratinized attached gingiva and hard palate, while the microbial communities of sub-gingival and supra-gingival plaque were distinct from others. The site-specialist hypothesis for oral microbiota was proposed that there was a prime habitat for oral microbiota where most of oral microorganisms grew and divided (Mark Welch et al., 2019). Besides, microbial compositions in the oral cavity and esophagus are similar but essentially different (Figure 1). Dong et al. (2018) collected oral samples from saliva, tongue dorsum and supragingival plaque, as well as esophageal samples from upper, middle and lower of the esophagus. There were 594 genera subjected to 29 phyla in the esophagus and 365 genera subjected to 29 phyla in the oral cavity. Both of them detected high relative abundances of bacteria, including *Streptococcus*, *Neisseria*, *Prevotella*, *Actinobacillus*, and *Veillonella*. The predominant genus in the esophagus was *Streptococcus*, while the predominant genus in the oral cavity was *Neisseria*.

It is well-established that oral microbiota has a close association with many oral diseases, such as periodontitis, tooth reduction, dental caries. In addition to these diseases, oral microbiota alteration has been suggested to play an important role in diabetes, rheumatoid arthritis, chronic obstructive pulmonary diseases, cardiovascular diseases, and cancer (Gupta et al., 2017; Bourgeois et al., 2019b; Sun et al., 2020; Tuominen and Rautava, 2021). In particular, the relative abundance of *Porphyromonas gingivalis* in patients with digestive tract cancer (tongue/pharyngeal cancer, EC, gastric cancer, colorectal cancer) was higher than that in healthy controls (Kageyama et al., 2019). Other studies have reported the relationship between oral microbiota and EAC. On the one hand, a prospective study showed that a history of periodontal disease and tooth loss was associated with a 43% and 59% increased risk of EAC over 22–28 years of follow-up (Lo et al., 2021). On the other hand, the salivary bacterial diversity was significantly higher in EC patients than that in healthy controls (Kageyama et al., 2019). And a case–control study in China showed a significant shift in oral microbiota between the EC patients and the healthy participants. By detecting salivary microbial communities, EC patients had a higher relative abundance of phylum *Firmicutes*, class *Negativicutes*, order *Selenomonadales*, family *Veillonellaceae*, and genus *Prevotella*, and a lower relative abundance of phylum *Proteobacteria*, class *Betaproteobacteria*, order *Neisseriales*, family *Neisseriaceae*, and genus *Neisseria* in contrast with healthy individuals (Zhao et al., 2020). Moreover, another research showed that BE patients had a higher relative abundance of *Firmicutes* and a lower relative abundance of Proteobacteria in saliva compared to patients without BE (Snider et al., 2018), which was in accordance with the EC patients. All of these researches support a link between oral microbiota and EAC development.

## Human Microbiota in Esophageal Adenocarcinoma

Determination of the variation in human microbiota between health and disease is crucial to understanding the biases that occur in disease. There were many studies of the esophageal microbiota alteration in EAC. One prior case–control study found altered microbial communities in esophageal carcinogenesis, notably increases in *Proteobacteria* and reductions in *Firmicutes*. Besides, two families, *Verrucomicrobiaceae* and *Enterobacteriaceae*, became increasingly in HGD and EAC (Snider et al., 2019). Similarly, Zaidi and colleagues found a high prevalence of *Escherichia coli* in EAC and BE patients, while it was lacking in the tumor adjacent normal epithelium. All these indicated that the shift toward *Enterobacteriaceae* in esophageal carcinogenesis was not accidental. According to the research of the esophageal microbiome, there is a reduction of *Streptococcus* and an increase of *Prevotella* in EAC compared with healthy controls (Lopetuso et al., 2020). Zhou and colleagues discovered a unique esophageal microbiota in EAC subjects. Compared with normal esophageal, there were abundant *Proteobacteria* and *Firmicutes*, mostly like *Staphylococcus aureus*, *Streptococcus infantis*, *Moryella* sp. and *Lactobacillus salivarius*, and rare *Actinobacteria* (*Rothia mucilaginosa*) in the EAC esophageal microbiota (Zhou et al., 2020). Most of them were lactic acid-producing bacteria. As is well established, sustained high lactate level could promote angiogenesis, immune escape, cell migration and metastasis, thus supporting the tumorigenesis and progression (San-Millán and Brooks, 2017). The authors proposed that increased lactic acid-producing bacteria in the esophageal may work as one of the factors contributing to the development of the EAC. Additionally, there is a high prevalence of *Candida albicans* and *Candida glabrata* in more than half of the human EAC samples (Zaidi et al., 2016), which suggests the existence of fungal microbiota in the esophagus.

Esophageal adenocarcinoma has been studied extensively in relation to the esophageal microbiota, but relatively insufficiency so with respect to microbiota at other sites of the human body. A prospective study examined the relationship between EAC and oral microbiota. In mouthwash samples, there was a high amount of *Tannerella forsythia*, *Actinomyces cardiffensis*, *Veillonella* oral taxon 917, and *Selenomonas* oral taxon 134 was associated with higher EAC risk, whereas a low amount of *Prevotella nanceiensis*, *Corynebacterium durum*, *Streptococcus pneumoniae*, *Lachnoanaerobaculum umeaense*, *Solobacterium moorei*, *Oribacterium parvum*, *Neisseria flavescens*, *Neisseria sicca*, and *Haemophilus* oral taxon 908 was associated with lower EAC risk (Peters et al., 2017). If these results turn out to characterize the shift with the progression of EAC, rather than simply correlative, they demonstrate potential prevention *via* protecting against microbial exposure.

## Human Microbiota in Gastroesophageal Reflux Disease and Barrett’s Esophagus

Gastroesophageal reflux disease was regarded as a risk factor for EAC and BE was established as the precursor lesion of EAC. It is of momentous significance to clarify the human microbiota of GERD and BE for the EAC researches. An early study by Yang in 2009 found the potential link between alterations in the human distal esophageal microbiome and reflux-related disorders. The bacterial communities of 34 patients were checked after biopsies of the distal esophagus by 16S rRNA gene sequencing. The authors classified the human esophageal microbiome into two types according to the results of gene analysis. The type I esophageal microbiome was more relevant to the normal esophagus, while the type II esophageal microbiome was more relevant to the abnormal esophagus. The type I microbiome had a higher mean abundance of *Streptococcus*, while the type II microbiome had a higher level of microbial diversity and a higher average proportion of Gram-negative bacteria. They also concluded that the type II microbiome was mainly composed of Gram-negative anaerobes or microaerophiles, including *Veillonella*, *Prevotella*, *Neisseria*, *Haemophilus*, *Rothia*, *Granulicatella*, *Campylobacter*, *Fusobacterium*, *Porphyromonas*, and *Actinomyces*. The predominant organisms shifted from Gram-positive aerobic bacteria to Gram-negative anaerobic bacteria (Yang et al., 2009).

**FIGURE 2:**
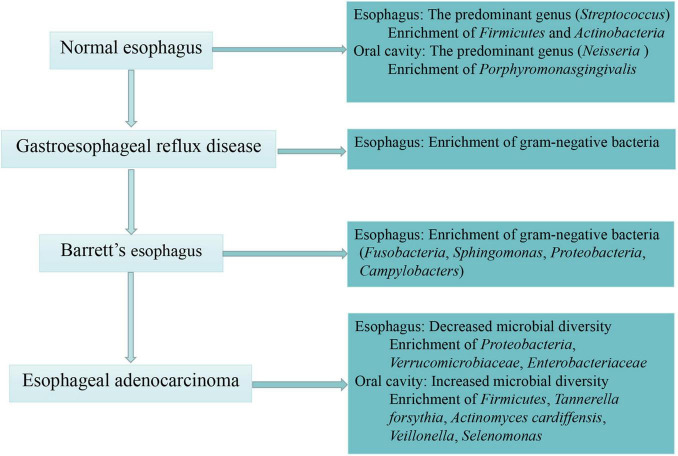
The process of esophageal carcinogenesis and associated human microbiota. This figure describes specific human bacterial alternations in the normal esophagus, esophageal adenocarcinoma and its precursor lesion—gastroesophageal reflux disease and Barrett’s esophagus.

Similarly, increasing evidence has supported a shift toward some specific Gram-negative bacteria in the EAC cascade. It was reported that Gram-negative organisms colonizing the esophageal mucosa, especially *Campylobacters*, became increasingly in GERD and BE compared with healthy control groups (Blackett et al., 2013). Other studies found that there was a shift away from *Firmicutes* and toward Gram-negative *Fusobacteria*, *Sphingomonas*, *Proteobacteria* and an unclassified species of *Campylobacter* in BE compared to controls (Snider et al., 2019; Zhou et al., 2020). Of note, Lopetuso and colleagues found that the relative abundance of *Streptococcus* and *Granulicatella* decreased in the EAC mucosa compared with BE mucosa, with the relative abundance of *Prevotella* increased correspondingly. The authors considered EAC as an extreme dysbiotic perturbation of microbiota in BE mucosa which consisted largely of Gram-negative bacteria (Lopetuso et al., 2020). In summary, alteration of the human microbiota in EAC cascade was presented as decreased microbial diversity and enrichment of Gram-negative bacteria in esophagus as well as increased microbial diversity and enrichment of *Firmicutes*, *Tannerella forsythia*, *Actinomyces cardiffensis* in oral cavity (Figure 2).

## Human Microbiome as Potential Diagnostic Biomarkers and Screening Tools for Esophageal Adenocarcinoma

Current screening tools have respective advantages and disadvantages. The gold-standard technique of EC and preinvasive lesions is endoscopy with adequate targeted biopsies. However, this method cannot be used extensively due to the time and expense. Esophageal tissue samples including sponges and inflatable balloons have good specificity but lack sensitivity (Lao-Sirieix and Fitzgerald, 2012). It has been confirmed some specific pathogens could promote the development of EC, while other pathogens could be a protective factor against the reduced risk of EC. As a result, some biomarkers have enormous potential as diagnostic biomarkers and screening tools for EAC (Table 1). Finding out a biomarker with excellent sensitivity and specificity is the key to extending the biomarker detection application field.

**TABLE 1:** Human microbiota studies for esophageal adenocarcinoma.

Study	Population(s)	Study sample size	Study period	Study platform	Sample type	Main findings	Tool type
Lopetuso et al., 2020	Rome	BE (*n* = 10); EAC (*n* = 6); controls (*n* = 16)	2020	16S rRNA	Esophageal mucosa	*Prevotella*, *Veillonella*, and *Leptotrichia* had higher abundance in EAC than that of CTRL, while *Streptococcus* had lower abundance.	Diagnosis
Zhou et al., 2020	Australia	RE (*n* = 20); BE (*n* = 17); EAC (*n* = 6); controls (*n* = 16)	2020	16S rRNA	Esophageal mucosa	Compared with CTRL, there was a reduction of *Actinobacteria* in EAC, with an increase of *Firmicutes* and *Proteobacteria*.	Diagnosis
Peters et al., 2017	America	EAC (*n* = 81); controls (*n* = 160)	2017	16S rRNA	Mouthwash samples	The abundances of species *Tannerella forsythia* were positive correlated with risk of EAC, while the abundances of the genus *Neisseria* and the species *Streptococcus pneumoniae* were inversely correlated with risk of EAC	Diagnosis
Snider et al., 2019	United States	LGD (*n* = 6); HGD (*n* = 5); BE (*n* = 14); EAC (*n* = 4); controls (*n* = 16)	2019	16S rRNA	Saliva samples	There was a shift toward *Enterobacteriaceae* and *Akkermansia muciniphila*, while away from *Firmicutes* in patients with HGD and EAC relative to controls	Diagnosis
Zhao et al., 2020	China	EC (*n* = 39); controls (*n* = 51)	2020	16S rDNA	Saliva samples	*Prevotella* was enriched in EC, while *Neisseria* was decreased.	Diagnosis
Peter et al., 2020	United Kingdom	IM (*n* = 10); LGD (*n* = 10); HGD (*n* = 10); EAC (*n* = 12); controls (*n* = 10)	2020	16S rDNA	Esophageal mucosa	The abundance of the phylum *Planctomycetes* and the archaean phylum *Crenarchaeota* in EAC was significantly lower than that in CTRL	Diagnosis
Deshpande et al., 2018	Australia	EoE (*n* = 1); GERD (*n* = 29); GM (*n* = 7); BE (*n* = 5); EAC (*n* = 1); CTRL (*n* = 59)	2018	16S rRNA; 18S rRNA; shotgun sequencing	Esophageal mucosa; esophageal brushings	An enrichment of Gram-negative bacteria associated with the oral cavity and microbial lactic acid production in the EAC cascade	Diagnosis
Elliott et al., 2017	United Kingdom	ND (*n* = 20); BE (*n* = 23); EAC (*n* = 19); CTRL (*n* = 20)	2017	16S rRNA	Esophageal mucosa; esophageal brushes; Cytosponge samples	*Lactobacillus fermentum* was enriched in EAC compared with controls	Diagnosis
Kawasaki et al., 2020	Japan	EC (*n* = 61); CTRL (*n* = 62)	2020	PCR	Subgingival dental plaque; saliva samples	The prevalence of *Tannerella forsythia*, *Streptococcus anginosus*, and *Aggregatibacter actinomycetemcomitans* was positively related to the presence of EC with high odds ratios, respectively	Diagnosis
Rajendra et al., 2013	Australia	BE (*n* = 77); BD (*n* = 35); EAC (*n* = 27); CTRL (*n* = 122)	2013	PCR; immunohistochemistry;	Esophageal mucosa; tumor specimens	High activity of *human papillomavirus* was strongly association with worse disease severity	Prognosis
Yamamura et al., 2016	Japan	EC (*n* = 325)	2016	PCR	Tumor specimens; tumor adjacent normal specimens	*Fusobacterium nucleatum* in EC was related to higher tumor stage and poor prognosis in the patients after the esophagus carcinoma resection	Prognosis

*BEM, esophageal metaplastic samples; BE, Barrett’s esophagus; IM, intestinal metaplasia; LGD, low-grade dysplasia; HGD, high-grade dysplasia; BD, Barrett’s dysplasia; EC, esophageal cancer; EAC, esophageal adenocarcinoma; CTRL, healthy control samples; PCR, polymerase chain reaction.*

With the increase in antibiotic treatment in the mid-twentieth century, infections of *Helicobacter pylori* began to decline, then the incidence of esophageal adenocarcinoma and eosinophilic esophagitis rises (May and Abrams, 2018). The large-scale pooled analysis found that *Helicobacter pylori* infection varied directly as the odds of BE and inversely proportional to the odds of GERD (Wang et al., 2018). However, Aghayeva and colleagues retrospectively analyzed cases in Azerbaijan, a high-prevalence region, and highlighted that there is no difference between the prevalence of *Helicobacter pylori* in BE and control group cases. The authors concluded that neither BE nor dysplasia is inversely associated with the prevalence of *Helicobacter pylori* (Aghayeva et al., 2019). Similarly, the hypothesis of the Swedish nationwide population-based cohort study was confirmed by calculating the standardized incidence ratios (SIRs), which were equal to the observed number of individuals in the *Helicobacter pylori* eradication cohort over the expected number of individuals in the Swedish background population. This study found that there is no evidence a gradually increased risk of BE or EAC is linked with *Helicobacter pylori* eradication treatment in spite of the increasing SIRs of BE and EAC after *Helicobacter pylori* eradication treatment (Doorakkers et al., 2020). Whether *Helicobacter pylori* infection influences EAC and its precursor is still a debatable point. However, it has been noted that *Helicobacter pylori* infection promotes Ki-67 expression in BE. According to a meta-analysis with 1243 samples, Ki-67 showed a reasonable diagnostic odds ratio of 5.54, sensitivity of 82% and specificity of 48% in identifying high-risk patients of EAC in BE group (Altaf et al., 2017). In addition to *Helicobacter pylori*, other human microbiota-associated biomarkers may be reasonably efficient in EAC screening and diagnosis. As a result of significantly increased abundance of *Prevotella* at the genus level and family level that covered all samples, Zhao and colleagues indicated that *Prevotella* was may be used in the early prediction or prevention of EC (Zhao et al., 2020). Overall, *Prevotella* and Ki-67 may play an important role in the personalized precision diagnosis of EAC.

Several studies have also implicated periodontal pathogens as potential diagnostic biomarkers for EAC. As mentioned above, Peters and colleagues indicated that *Tannerella forsythia* was strongly related to EAC. They observed that the increased abundance of *Tannerella forsythia* was correlated to the higher risk of EAC, while the decreased abundance of Neisseria and *Streptococcus pneumoniae* was correlated to the lower risk of EAC (Peters et al., 2017). Similarly, the prevalence of *Tannerella forsythia* and *Aggregatibacter actinomycetemcomitans* with EC patients was significantly higher in the subgingival plaque compared with healthy controls (Kawasaki et al., 2020). It is now well accepted that both *Tannerella forsythia* and *Aggregatibacter actinomycetemcomitans* are Gram-negative periodontal pathogens that might contribute to the pathogenesis of periodontitis (Sharma, 2010; Gholizadeh et al., 2017). A previous study suggested a possible correlation between *Aggregatibacter actinomycetemcomitans* and the increasing risk of pancreatic cancer (Fan et al., 2018). An oral microbiome-based model containing a relative abundance of *Streptococcus*, *Lautropia*, and *Bacteroidales* discriminated between BE patients and controls with the ROC of 0.94, the sensitivity of 96.9%, and the specificity of 88.2% (Snider et al., 2018). Our findings suggest that periodontal pathogens, like *Tannerella forsythia* and *Aggregatibacter actinomycetemcomitans*, may be utilized as biomarkers for detecting EAC-associated changes in the human microbiota.

## Human Microbiota for Clinical Prognosis Analysis of Esophageal Adenocarcinoma Patients

The late presentation of symptoms and the aggressiveness of EAC results in poor prognosis (Coleman et al., 2018). Interestingly, *human papillomavirus* (HPV)-related biomarkers in pre-cancer lesions can become an important prognostic indicator of EAC. A previous study has demonstrated that head and neck squamous cell carcinoma (HNSCC) patients with HPV-positive have a higher rate of overall survival and a lower risk of recurrence compared with HPV-negative patients (Ragin and Taioli, 2007; O’Rorke et al., 2012). Given the well-established impact that HPV status has on the prognosis of HNSCC, it is highly plausible that HPV-related EAC would show a similar prognosis. A prospective study has identified that high-risk HPV with transcription activity is associated with BD and EAC. Biopsy samples were used for HPV DNA determination *via* PCR and viral transcriptional activity determination *via* E6/7 oncogene mRNA expression and p16^INK4a^ immunohistochemistry. Compared with BE and controls, the proportion of HPV DNA-positive, p16^INK4a^ positivity and oncogene expression in Barrett’s dysplasia (BD) and EAC was significantly higher (Rajendra et al., 2013). The authors emphasized that HPV was strongly relevant to BD and EAC but irrelevant to BE and controls, which suggested the role of HPV in the pathogenesis of tumors. Based on preliminary studies, a retrospective case–control study assessed HPV-related biomarkers [retinoblastoma protein (pRb), cyclin D1 (CD1), Ki-67, and minichromosome maintenance protein (MCM2)] to estimate the prognostic value on the patients with BD and EAC. The authors found low expression of CD1 with a good prognosis in EAC (Rajendra et al., 2020). In contrast to HPV-negative patients, HPV-positive patients with low expression of CD1, high expression of MCM2, low expression of pRb, high expression of p16 and positive status of E6 and E7 mRNA had improved disease-free survival, suggesting HPV-positive EAC and HPV-negative EAC are two distinct diseases, exactly as in HNSCC (Rajendra et al., 2018).

Recently many studies about the relationship between *Fusobacterium nucleatum* and gastroenteric cancer have been reported. Yamamura and colleagues found the new application of *Fusobacterium nucleatum* DNA status in prognosis prediction in EC. The relative amounts of *Fusobacterium nucleatum* DNA were significantly higher in tumor tissue compared with adjacent normal tissue. The cancer-specific survival and OS were significantly shorter in *F. nucleatum*-positive individuals than that in *Fusobacterium nucleatum*-negative individuals. Similarly, the cancer-specific mortality was significantly higher in *Fusobacterium nucleatum*-positive individuals than that in *Fusobacterium nucleatum*-negative individuals. Thus, we consider this periodontal bacteria can be used for the clinical prognosis of the EC as an indicator (Yamamura et al., 2016).

A Japanese study has revealed that the presence of oropharyngeal allopatric flora was an independent predictive factor of post-esophagectomy pneumonia. The authors divided 675 patients into three groups by categorization of oropharyngeal flora, including indigenous flora (Ind group), antibiotic-sensitive microbes only (Allo-S group) and antibiotic-resistant microbes (Allo-R group). Compared with the Ind group, the incidence of postoperative pneumonia in the Allo-S and Allo-R groups increased markedly and the survival in the Allo-R group significantly decreased (Yuda et al., 2020). Hence, it is anticipated that we can prevent post-esophagectomy pneumonia from the classification of the oral microbiome someday.

## Potential Mechanisms of Microbe-Mediated Esophageal Carcinogenesis

**FIGURE 3:**
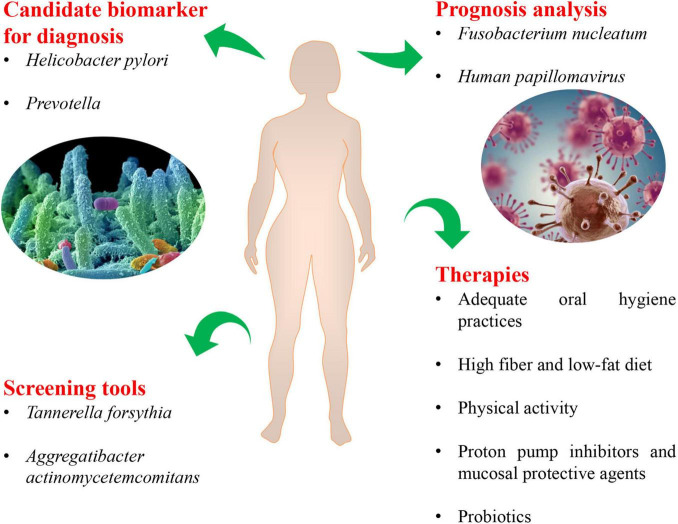
The molecular mechanisms of microbe-mediated in EAC carcinogenesis. On the basis of the known contribution of human microbiota in esophageal carcinogenesis, the main mechanisms included inflammation, metabolism, and genotoxicity. Alteration of human microbiota in EAC showed a shift toward Gram-negative bacteria. Some specific pathogens, such as *Campylobacter concisus*, *Helicobacter pylori*, and *Escherichia coli*, involved in the process of inflammation and EAC cascade by regulating the expressions of toll-like receptors (TLRs) and pro-inflammatory mediators such as TNF-α, IL-18, COX-2, prostaglandins. And the components of Gram-negative bacteria activated the NLRP3 inflammasome and NF-κB pathway. Besides, detrimental metabolites, such as hydrogen sulfide, products of protein fermentation and bile acid metabolism, could play an important role in the initiation and progression of EAC. Some Gram-negative bacteria produced the cytolethal distending toxin (CDT), which could induce DNA damage and trigger EAC carcinogenesis. LPS, lipopolysaccharides; NF-κB, nuclear factor kappa B; TLRs, toll-like receptors; CDT, cytolethal distending toxin; TNF-α, tumor necrosis factor-α; IL, interleukin; COX, cyclooxygenase; PG, prostaglandin; p53, tumor protein 53; NLRP3, nucleotide-binding domain and leucine-rich repeat-containing protein 3.

The molecular mechanisms by which the human microbiota could initiate and drive tumorigenesis have always been the focus. Genomic integration, genotoxicity, inflammation, immunity and metabolism are major mechanisms (Lv et al., 2019; Scott et al., 2019). Given the well-established impact that the composition of human microbiota and its activity mediated inflammation and genotoxicity in tumorigenesis of many cancers, such as colon cancer, liver cancer and pancreatic cancer (Cani and Jordan, 2018), many investigators were making their attempts to elucidate the mechanism of human microbiota during carcinogenesis of EC, including metabolites, genotoxicity, inflammation and immune dysregulation. Here, we review the main microbiota-associated mechanisms which have been under extensive research in esophageal carcinogenesis (Figure 3). However, despite considerable evidence to suggest significant changes in human microbiota following EC, it remains to be determined whether these changes have a causal effect or are only correlative in nature.

### The Human Microbiota, Inflammation and Esophageal Carcinogenesis

#### Specific Pathogens

Sustained infection or non-infectious factors may lead to various pro-inflammatory and oncogenic mediators in the process of chronic inflammation, eventually resulting in tumor promotion (Khandia and Munjal, 2020). Data from several studies suggested that *Campylobacters* may play an important role in the process of inflammation and esophageal carcinogenesis. With the dominant change of the appearance of *Campylobacters* during the disease states, Blackett and colleagues found IL-18 expression significantly increases in both GERD and BE colonized subjects compared with non-colonized subjects (Blackett et al., 2013). IL-18 is a multifunctional cytokine that induces pro-inflammatory cytokine expression and is associated with anti-tumor immunity. Many studies implicate that the serum IL-18 levels of EC patients were significantly higher than the control group, deficiency of IL-18 can aggravate the progression and development of EC and IL-18 signaling is strongly associated with BE and EAC (Diakowska et al., 2006; Babar et al., 2012; Li et al., 2018). The *Campylobacters* consist almost entirely of *Campylobacter concisus*, which virtually only appearing in the disease states. The cell culture model of Barrett’s cell lines reported a marked increase and a time-dependent manner in the expression of pro-inflammatory mediators (IL-18 and TNF-α) and tumor suppressor gene (p53) in co-culture with *Campylobacter concisus* (Mozaffari Namin et al., 2015b). By means of a comprehensive analysis of *Campylobacter* species, a new viewpoint that *Campylobacter* species modulated the host inflammatory response, and then, it initiated the EAC cascade was presented theoretically (Kaakoush et al., 2015). Previous research has indicated that the colonization of *Helicobacter pylori* in the esophagus increased the incidence of BE and EAC (Liu et al., 2011). Subsequently, some researchers have implicated the possible role of *Helicobacter pylori* in the malignant progression of the esophagus by promoting the expression of gastrin, COX-2, prostaglandins and Ki-67 (Kountouras et al., 2019; Doorakkers et al., 2020). Similarly, investigators also explained the association between *Enterobacteriaceae* infection and esophageal carcinogenesis has been proposed. The expression of toll-like receptors (TLRs) 1–3, 6, 7, and 9 significantly increases in EAC rats (Zaidi et al., 2016), demonstrating an *Escherichia coli*-related esophageal carcinogenesis. As mentioned previously, *Campylobacters*, *Helicobacter pylori* and *Escherichia coli* seemed to be specifically involved in EAC cascade through pro-inflammatory cytokine expression. Nevertheless, it is not yet clear whether there is a causality between specific pathogens and EAC.

#### Microbial Metabolites

In addition to specific pathogens, human microbiota could trigger carcinogenesis as an integrated community. A quintessential example should be cited that microbiota dysbiosis and host–microbiota interactions seemed to promote colorectal tumorigenesis (Schwabe and Jobin, 2013). The metabolites play an important role in the initiation and progression of cancer. Protective metabolites are represented by short-chain fatty acids. And detrimental metabolites are represented by hydrogen sulfide, products of protein fermentation and bile acid metabolism (Louis et al., 2014). Evidence suggests that human microbiota contributes to esophageal tumorigenesis, not only *via* the inflammation of specific pathogens but also *via* the influence of its metabolome (Louis et al., 2014). Some bacteria produced certain compounds which might be a carcinogen. Bile acid metabolism is one of the most important microbial metabolism. Researchers found that chronic exposure to bile acids might result in esophageal carcinogenesis through over-expression of glucose-6-phosphate dehydrogenase and active nuclear factor-kB (NF-κB) (Munemoto et al., 2019). The toll-like receptor-4 ligand, named LPS, is produced by Gram-negative bacteria. LPS can activate the NOD-like receptor protein 3 inflammasome and NF-κB pathway. The esophageal microbiome, dominated by Gram-negative bacteria, might contribute to materializing the inflammation-mediated carcinogenesis in BE by LPS *via* relaxing the lower esophageal sphincter and delaying gastric emptying (Yang et al., 2012; Nadatani et al., 2016; Lv et al., 2019). This provides new evidence about the molecular mechanisms underlying the association between LPS and esophageal carcinogenesis.

### The Human Microbiota, Genotoxicity and Esophageal Carcinogenesis

Genotoxicity refers to structural DNA damage (Scott et al., 2019). A multitude of Gram-negative bacteria mainly including *Escherichia coli*, *Actinobacillus actinomycetemcomitans*, Campylobacters and *Helicobacter pylori* could produced the cytolethal distending toxin (CDT), which could induce DNA damage and promote cancer (Nesiæ et al., 2004; He et al., 2019). Certain species within *Enterobacteriaceae* produced a DNA-alkylating genotoxin so that led to DNA damage, which might accelerate tumor progression (Wilson et al., 2019). *Helicobacter pylori* is prescribed for class I carcinogen. *Helicobacter pylori* toxin cytotoxin-associated gene A induced oxidative DNA damage and modulated the host inflammatory response in gastric carcinogenesis (Wroblewski et al., 2010). It continues to be controversial whether *Helicobacter pylori* influences the canceration course of esophageal. The study of esophageal epithelial cell transfection has demonstrated that *Helicobacter pylori* infection led to the up-regulated expression of microRNA-212-3p targeted COX2 and miR-361-3p targeted CDX2 through the translation inhibition of miRNAs, which contributed to the phenotypic transformation of esophageal epithelial cells (Teng et al., 2018).

## The Human Microbiota-Based Therapies in Esophageal Cancer

The therapeutic principle of esophageal cancer is based on individualized comprehensive treatment. In fact, surgery combined with radiotherapy and chemotherapy has become the mainstay of clinical treatment for EC (Stahl et al., 2010). It is now well established that several healthy behaviors are helpful for cancer prevention, including a healthy diet, physical activity, weight control and alcohol consumption limit (Rock et al., 2020). In addition, some interventions related to the altered human microbial composition may become the new adjuvant treatment in EC, such as proton pump inhibitors, probiotics, mucosal protective agents, and chlorhexidine mouth rinse.

### Oral Hygiene

A large body of published research has consistently demonstrated poor oral hygiene was associated with a higher risk of cancers, such as oral cancer (Deng et al., 2021), gastric cancer (Zhang et al., 2021), colorectal cancer (Wang et al., 2021). Based on the outcomes of two case–control studies, poor oral hygiene was an important risk factor for EC (Mmbaga et al., 2020; Poosari et al., 2021). And patients who received dental prophylaxis had a reduced risk of EC (Lee et al., 2014). The data highlighted the importance of adequate oral hygiene practices, which could be a simple means to prevent various cancers (Yano et al., 2021). The interdental brush is a form of toothbrush which could be inserted between the teeth in order to remove plaque (Worthington et al., 2019). Denis and colleagues demonstrated that toothbrushing and interdental brushing can decrease the number of oral bacteria in particular those who were associated with periodontal disease (Bourgeois et al., 2019a). The individuals may benefit from the daily use of toothbrushing and interdental brushing. Previous research has argued that interdental brush reduces interdental bleeding compared with manual toothbrush (Bourgeois et al., 2016). As for the frequencies of toothbrushing, it is suggested that toothbrushing twice daily for 2 min in order to prevent periodontal disease (Sälzer et al., 2020). Additionally, a randomized controlled trial analyzed the oral and esophageal microbiota and gene expression of the esophagus before and after treatment of chlorhexidine mouth rinse. The authors identified significant alterations in the oral and esophageal microbiota and demonstrated that the alterations of the esophageal microbiota could be closely related to changes in gene expression of the esophagus, suggest the clinical application of mouth rinse treatment in EC (Annavajhala et al., 2020).

### Diet

Diet and nutrition are the major areas of interest within the prevention of chronic diseases and cancer. A healthy diet should include nutritious food, whole grains, fiber-rich legumes, a variety of vegetables and fruits (Rock et al., 2020). Several dietary patterns are representative, including Mediterranean, Dietary Approaches to Stop Hypertension, Okinawa and vegetarian diets. Many bioactive nutrients of these diets have played an effective role in the epigenetic modification and maintaining the balance of intestinal microbiota (Divella et al., 2020). Mediterranean diet (MD) is internationally regarded as a “long life” diet (Mentella et al., 2019; Martinon et al., 2021). The composition of Okinawa and Dietary Approaches to Stop Hypertension diets is similar to MD. Recent studies have shown that MD is associated with a decreased cancer mortality risk (Molina-Montes et al., 2020). Analyses indicated the decreased risk of gastro-intestinal cancer was associated with a vegetarian diet (Tantamango-Bartley et al., 2013). A low-fiber, high-fat, and high-refined-sugar diet might be responsible for the declining diversities (Lloyd-Price et al., 2016). Nevertheless, diet therapy is expected to be universally accepted low-risk and patient-friendly intervention to prevent chronic diseases and even cancer among the population, just as vaccines prevent flu. New therapeutic strategies of the EC could be proposed by targeted dietary intervention. Enteric pathogenic bacteria boosted their growth and pathogenicity by exploiting some short-chain fatty acids, microbiota-derived sources of carbon, and other nutrients (Bäumler and Sperandio, 2016). Host diet has a profound effect on the composition of the gut microbiota and its metabolites. Nobel and colleagues found a negative correlation between fiber intake and the relative abundance of Gram-negative bacteria, most notably *Betaproteobacteria* (Nobel et al., 2018). This study provided new evidence about the potential mechanisms underlying the association between dietary fiber and esophageal microbiome composition. Current consensus suggests that the risk of EAC could decrease after a reduction in total dietary fat, saturated fat, and cholesterol (Thrumurthy et al., 2019). Data from a study suggested that participants with reduced microbial gene richness presented more higher aberrant metabolism and low-grade inflammation, and weight-loss dietary intervention may succeed in improving these changes (Cotillard et al., 2013). Similarly, Münch and colleagues indicated that a high-fat diet led to the alterations of gut microbiota which accelerated inflammation and esophageal carcinogenesis in the mouse model which was irrelevant to obesity (Münch et al., 2019). In the future, a high fiber and low-fat diet may be helpful to prevent EC.

### Physical Activity

Several studies have documented that exercise contributes to the human gut microbiota alternation (Shukla et al., 2015; Allen et al., 2018). Long-term regular exercise lead to higher diversity and significant shifts of major bacterial taxa in human gut microbiota, especially a higher relative abundance of the genus *Akkermansia* (Clarke et al., 2014). In addition, the role of physical activity in cancer prevention has received increased attention across a number of disciplines in recent years. There are consistent evidence that physical activity plays an important role in preventing cancer. An American roundtable report found that physical activity can reduce the risk of seven types of cancer including EAC (Patel et al., 2019). Aerobic exercise and muscle strength training before esophagectomy is useful for reducing the rates of postoperative respiratory complications in EC patients (Akiyama et al., 2021). Based on the preventive effect of exercise on EC, a study set it out to investigate the usefulness of evaluating the prognosis. The 6-min walking distance is a clinical examination gradually used to evaluate the prognosis of patients after surgery. It has the advantages of low cost and easy implementation. A retrospective cohort study has established that the 6-min walking distance is directly proportional to the overall survival in patients undergoing esophagectomy (Kondo et al., 2021).

### Proton Pump Inhibitors and Mucosal Protective Agents

Proton pump inhibitors (PPIs) are used extensively for the full spectrum of gastric-acid-related diseases in clinic (Malfertheiner et al., 2017). It inhibits the activity of gastric H^+^/K^+^-adenosine triphosphatase, resulting in the inhibition of acid secretion from parietal cells (Rochman et al., 2021). Previous researches have established that long-term PPI use induces changes in the gut microbiota (Clooney et al., 2016; Malfertheiner et al., 2017). Compared with controls not using PPI, PPI users had decreased relative abundance of Gram-negative bacteria and increased relative abundance of *Streptococcus* (Snider et al., 2019). Changes in esophageal microbiota were observed before and after 8 weeks of PPI treatment. The predominant decreased taxa was *Comamonadaceae*, while the main increased taxa *were Clostridiaceae*, *Lachnospiraceae*, *Micrococcaceae*, *Actinomycetaceae*, *Gemellales*. As we discussed above, there was a shift toward Gram-negative bacteria in the EAC cascade. Although there is no direct evidence, PPI treatment may potentially benefit the patients with esophageal precancerous lesions (Amir et al., 2014). Similarly, mucosal protective agents are also applied extensively in the treatment of the gastric diseases (Haruma and Ito, 2003). In a murine Eosinophilic esophagitis (EoE) model, supplementation with *Lactococcus lactis* NCC 2287 attenuated esophageal eosinophilic inflammation (Holvoet et al., 2016). Recent research in a rat model suggests that rebamipide, a mucosal protective agent, can reduce BE development and alter the esophageal microbiome composition, in particular *Lactobacillus* and *Clostridium* (Kohata et al., 2015).

### Probiotics

Probiotic is a major area of interest within the field of microbiotic therapy. Probiotics therapeutic tests showed a significant inhibitory effect on the expression of biomarkers that contribute to BE transformation and indicated the possibility for the prevention of BE to EAC (Mozaffari Namin et al., 2015a). Besides, probiotics can be used to modulate the human microbiota in postoperative patients. A prospective trial evaluated the effect of probiotics on the prognosis of postoperative patients with EC (Lina et al., 2018). The result suggested that probiotics can reduce the rates of abdominal distension, constipation and gastric retention in postoperative patients with esophageal cancer.

## Conclusion

**FIGURE 4:**
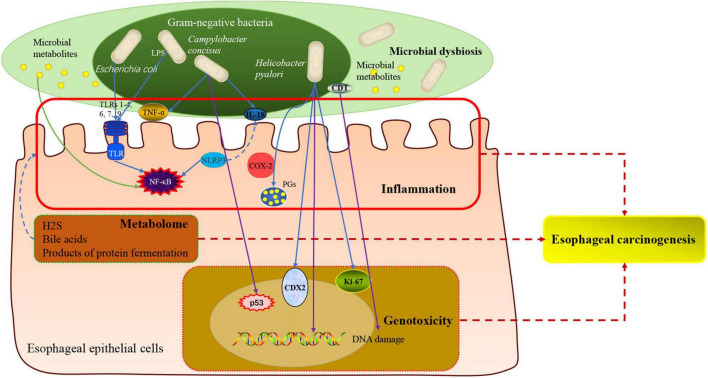
The novel microbiota-related targets in screening, diagnosis, prognosis, and therapy for esophageal adenocarcinoma.

The manuscript briefly summarizes our current knowledge regarding the relationship between human microbiota and the esophageal adenocarcinoma cascade. And it brings thinking from the fields of prevention, diagnosis, prognosis, and therapy for EAC (Figure 4). The current findings have identified decreased microbial diversity and altered human microbial communities in esophageal carcinogenesis, especially *Enterobacteriaceae*, *Campylobacters*, and acid-producing bacteria, periodontal pathogens (*Tannerella forsythia* and *Aggregatibacter actinomycetemcomitans*). *Helicobacter pylori*, *Prevotella*, *Tannerella forsythia*, and *Aggregatibacter actinomycetemcomitans* may be utilized as biomarkers for personalized precision diagnosis and screening of EAC. The expression of HPV-related biomarkers, the classification of the oral microbiome, and *Fusobacterium nucleatum* DNA status can become an important prognostic indicator of EAC. Notably, novel clinical interventions related to the human microbiota may also be used to treat EC, including adequate oral hygiene practices, a high fiber and low-fat diet, physical activity, PPI, mucosal protective agents and probiotics, which might benefit patients significantly. From this review, it emerged clearly that the human microbiota may impact the initiation and progression of EAC since it not only mediates inflammation and genotoxicity as specific pathogens, but also triggers detrimental metabolites as an integrated community. All mechanisms are not mutually exclusive and may be involved in tumorigenesis in a stage-specific and case-specific manner (Chen et al., 2017).

There were also certain limitations. Although preliminary studies have provided a comprehensive view of the role of the human microbiota in EAC development, information on causative effects on EAC cascade remained to be elucidated. This area needs more research to truly understand the complex mechanisms behind the impact of the human microbiota on tumorigenesis of EAC.

## Author Contributions

FP conceptualized the study, revised the manuscript, and supervised the study. WD drafted the manuscript and made the figures. LP and BY collected the literature and revised the manuscript. ZL improved the manuscript. All authors read and approved the final manuscript.

## Conflict of Interest

The authors declare that the research was conducted in the absence of any commercial or financial relationships that could be construed as a potential conflict of interest.

## Publisher’s Note

All claims expressed in this article are solely those of the authors and do not necessarily represent those of their affiliated organizations, or those of the publisher, the editors and the reviewers. Any product that may be evaluated in this article, or claim that may be made by its manufacturer, is not guaranteed or endorsed by the publisher.
